# Mechanism of Platelet‐Rich Plasma in Promoting Diabetic Wound Healing via the PI3K/AKT Signaling Pathway to Regulate Collagen Synthesis and Angiogenesis

**DOI:** 10.1155/jdr/1254597

**Published:** 2026-04-20

**Authors:** Hongyan Liu, Wenzhen Huang, Shuting Jiang, Beizhan Yan, Cunquan Kong, Weiyan Zhu, Qi Xie, Cuiyun Cui

**Affiliations:** ^1^ Department of Transfusion Medicine, Henan Provincial People′s Hospital, People′s Hospital of Zhengzhou University, Zhengzhou, China, zzu.edu.cn; ^2^ Department of Endocrinology, Henan Provincial People′s Hospital, People′s Hospital of Zhengzhou University, Zhengzhou, China, zzu.edu.cn; ^3^ Department of Pathology, Henan Provincial People′s Hospital, People′s Hospital of Zhengzhou University, Zhengzhou, China, zzu.edu.cn

**Keywords:** angiogenesis, diabetic wound healing, fibroblast proliferation, PI3K/AKT signaling pathway, platelet-rich plasma (PRP), TGF-*β*/SMAD2 signaling

## Abstract

Platelet‐rich plasma (PRP) has been found effective in wound healing, yet the underlying mechanisms in the healing of diabetic wounds remain unclear. In this study, we focus on investigating the role of PRP in enhancing the wound healing in a diabetic mouse model, specially through the modulation of the PI3K/AKT signaling pathway and its effect on collagen production and angiogenesis in the wounds of diabetic mouse model. In our in vitro experiments, we observed that PRP, in a concentration‐dependent manner, significantly stimulated the proliferation, migration, and collagen synthesis in human dermal fibroblasts. Concurrently, PRP activated the PI3K/AKT and TGF‐*β*/SMAD2 signaling pathways. In vivo, utilizing a diabetic mouse model of wound healing, PRP treatment significantly enhanced wound healing by accelerating wound closure, improving collagen deposition and organization, and promoting angiogenesis, as evidenced by increased expression of CD31 and VEGF. Importantly, the application of the PI3K inhibitor LY294002 effectively inhibited the observed effects of PRP. Additionally, the activation of the TGF‐*β*/SMAD2 signaling pathway induced by PRP was also suppressed when PI3K was inhibited, suggesting that the PI3K/AKT pathway functions upstream to regulate TGF‐*β*/SMAD2 signaling. These findings elucidate the role of PRP in facilitating diabetic wound healing through the activation of the PI3K/AKT pathway, which subsequently cross‐regulates TGF‐*β*/SMAD2 signaling, thereby enhancing cellular functions vital for tissue regeneration. Our results contribute valuable mechanistic insights into the therapeutic potential of PRP in addressing impaired wound healing in diabetic patients.

## 1. Introduction

The challenge of impaired wound healing represents a significant complication associated with diabetes mellitus, frequently resulting in chronic ulcers, heightened infection risk, and a substantial likelihood of amputation [1–3]. This pathological condition is marked by a series of cellular dysfunctions, including reduced fibroblast proliferation, inadequate collagen synthesis, impaired angiogenesis, and sustained inflammation [4]. Collectively, these deficiencies disrupt the normal phases of tissue repair, creating a considerable clinical burden and highlighting the urgent need for the development of more effective therapeutic strategies [5].

In recent years, platelet‐rich plasma (PRP), an autologous concentrate rich in platelets and growth factors, has emerged as a promising biological intervention to enhance the healing process in diverse clinical contexts [6–8]. Its capacity to promote cellular proliferation, migration, and extracellular matrix (ECM) deposition has attracted significant interest within the field of regenerative medicine [9–11]. The therapeutic efficacy of PRP in wound healing is primarily attributed to its ability to release critical growth factors, such as platelet‐derived growth factor (PDGF), transforming growth factor‐beta (TGF‐*β*), and vascular endothelial growth factor (VEGF) upon activation [12]. These factors are known to modulate key signaling pathways that regulate the cellular activities essential for effective tissue repair.

Cellular activities that are critical for the repair of tissues are influenced by various signaling pathways. Among these, the phosphoinositide 3‐kinase/protein kinase B (PI3K/AKT) pathway is recognized as a fundamental regulator of cell survival, proliferation, and migration [13–15]. Simultaneously, the TGF‐*β*/SMAD2 pathway is crucial in facilitating the differentiation of fibroblasts into myofibroblasts and in enhancing the synthesis of ECM components, particularly collagen [16].

Although the activation of these pathways by specific growth factors is extensively documented, the intricate mechanisms through which PRP, as a multifaceted biological mixture, coordinates these signals to expedite diabetic wound healing has not been thoroughly elucidated. In particular, the potential interactions or cross‐regulatory effects between the PI3K/AKT and TGF‐*β*/SMAD2 pathways in response to PRP stimulation represent a significant gap in current understanding, thereby hindering the rational optimization of PRP‐based therapeutic strategies. We postulated that PRP enhances diabetic wound healing by simultaneously activating both the PI3K/AKT and TGF‐*β*/SMAD2 signaling pathways, suggesting that these pathways do not operate independently but instead engage in a synergistic interplay to bolster tissue regeneration. To evaluate this hypothesis, we developed a comprehensive study aimed at investigating the effects of PRP on human dermal fibroblasts (HDFs) in vitro, followed by validation of the results in an in vivo diabetic mouse wound model.

The results obtained from this investigation reveal that PRP significantly enhances fibroblast functionality and activates both the PI3K/AKT and TGF‐*β*/SMAD2 signaling pathways. Notably, the in vivo inhibition of PI3K/AKT not only negated the positive impacts of PRP on wound closure and matrix formation but also diminished the activation of the downstream TGF‐*β*/SMAD2 pathway. This research provides insights into a novel mechanistic framework wherein PRP‐induced PI3K/AKT signaling acts as a pivotal upstream regulator that amplifies TGF‐*β*/SMAD2 activity, thus offering a more cohesive understanding of how PRP orchestrates various cellular processes to promote the healing of diabetic wounds.

## 2. Materials and Methods

### 2.1. Preparation and Characterization of PRP

Peripheral blood (50 mL) was collected from healthy volunteers by venipuncture into sodium citrate anticoagulant tubes (9:1 blood‐to‐citrate ratio). After gentle inversion to mix, PRP was prepared via a two‐step centrifugation protocol using a temperature‐controlled centrifuge. The first centrifugation was performed at 510 × g for 10 min at 20°C. The supernatant along with the buffy coat was transferred into a sterile tube, followed by a second centrifugation at 2220 × g for 20 min at 20°C. The lower platelet‐rich layer was retained, and excess supernatant was removed to achieve the target concentration. Platelet counts were determined using a hematology analyzer. The baseline whole‐blood platelet concentration was 259 × 10^9^/L, and the concentrated PRP reached 1312 × 10^9^/L, corresponding to a 5.01‐fold increase. For growth factor release, PRP was activated by adding thrombin–calcium gluconate activator (100 U/mL) at a 1:10 (activator:PRP) ratio. After incubation at 37°C for 30 min, samples were centrifuged at 2220 × g for 20 min. The supernatant was collected, and concentrations of PDGF‐AA, TGF‐*β*, and VEGF were measured using commercial ELISA kits according to the manufacturer′s instructions, with absorbance read at 450 nm on an automated microplate reader. In freshly prepared PRP (Day 0), growth factor levels were as follows: PDGF‐AA, 49.68 pg/mL; TGF‐*β*, 307.61 ng/mL; and VEGF, 123.82 ng/mL.

### 2.2. Cell Culture

HDFs were maintained in high‐glucose Dulbecco′s Modified Eagle Medium (DMEM, Gibco, United States) supplemented with 10% fetal bovine serum (FBS) and 1% penicillin/streptomycin at 37°C in a 5% CO2 incubator. All treatment groups, including the 0% PRP control, were cultured in medium supplemented with 10% FBS.

### 2.3. Functional Assays for Cell Proliferation

CCK‐8 Assay: HDFs were seeded in 96‐well plates and treated with the selected PRP concentrations. After 24 and 48 h of incubation, cells were incubated with a medium containing 10% CCK‐8 reagent (Vazyme, China) for 2–4 h. Absorbance was measured at 450 nm using a microplate reader. EdU Assay: DNA synthesis was evaluated using a commercial EdU kit (RiboBio, China) following the manufacturer′s instructions. EdU‐positive cells were visualized and quantified using fluorescence microscopy. Colony Formation Assay: HDFs were seeded at low density, treated with PRP, and cultured for 14 days. Colonies were fixed with methanol, stained with 0.1% crystal violet, and counted. Colonies containing more than 50 cells were considered viable.

### 2.4. Functional Assays for Cell Migration

Transwell Migration Assay: Serum‐starved HDFs were seeded into the upper chambers of Transwell inserts (Corning, United States). PRP‐containing medium was added to the lower chamber as a chemoattractant. After 24 h, cells that migrated to the lower surface were fixed, stained with crystal violet, and counted under a microscope. Wound Healing (Scratch) Assay: A confluent monolayer of HDFs was scratched using a sterile 200‐*μ*L pipette tip. After washing, cells were incubated with PRP‐containing medium. Wound closure was photographed at 0 and 48 h postscratch, and the percentage of wound closure was analyzed using ImageJ software.

### 2.5. Assessment of Collagen Synthesis

Hydroxyproline (HYP) content measurement: The HYP content in the cell culture supernatant was quantified using a commercial Hydroxyproline Assay Kit (Abbkine, China, Cat# KTB1490) following the manufacturer′s protocol. Absorbance was read at 560 nm. Reverse Transcription‐quantitative PCR (RT‐qPCR) for collagen genes: Total RNA was extracted from HDFs using TRIzol reagent (Invitrogen, United States). cDNA was synthesized using a reverse transcription kit (Vazyme, China). The mRNA expression levels of collagen Type I alpha 1 chain (COL1A1) and collagen Type III alpha 1 chain (COL3A1) were analyzed using SYBR Green PCR Master Mix (Vazyme, China) on a qTOWER3 G real‐time PCR system (Analytikjena, Germany). GAPDH served as the internal control.

### 2.6. Western Blot

Total cellular proteins were extracted using RIPA lysis buffer containing protease and phosphatase inhibitors (Beyotime, China). Protein concentration was determined using a BCA Protein Assay Kit (Beyotime, China). Equal amounts of protein were separated by SDS‐PAGE and transferred onto PVDF membranes (Millipore, United States). After blocking with 5% nonfat milk, membranes were incubated overnight at 4°C with primary antibodies. The antibodies targeted the following proteins: TGF‐*β*, p‐SMAD2, p‐PI3K, p‐AKT, and GAPDH (all from Proteintech Group, China). After incubation with HRP‐conjugated secondary antibodies, protein bands were visualized using an ECL detection kit (Thermo Fisher Scientific, United States) and analyzed using ImageJ software.

### 2.7. Animal Model and Experimental Design

Diabetic Foot Ulcer (DFU) Model: Male C57BL/6 mice (6 weeks old, *n* = 30) were purchased from SPF (Beijing) Biotechnology Co. Ltd. Diabetes was induced by five consecutive daily intraperitoneal injections of streptozotocin (STZ, Sigma, United States) at 50 mg/kg in citrate buffer. Mice with blood glucose levels ≥ 16.7 mmol/L 1 week after the last injection were considered diabetic. Full‐thickness skin wounds (6 mm in diameter) were created on the dorsum of the foot of anesthetized diabetic mice. Experimental Groups: Mice were randomly divided into five groups (*n* = 6 per group): (1) Negative control (saline, topically every 3 days), (2) PRP group, (3) PRP + DSMO group, and (4) PRP + LY294002 group (PRP applied topically every 3 days).

### 2.8. In Vivo Efficacy Evaluation

Wound Healing Dynamics: Digital photographs of wounds were taken on Days 0, 3, 6, and 9 postwounding. The wound area was calculated using ImageJ software, and the wound closure rate was expressed as a percentage of the original area. Histological Analysis: On Day 9, wound tissues were harvested, fixed in 4% paraformaldehyde, paraffin‐embedded, and sectioned. H&E Staining: Sections were stained with hematoxylin and eosin to evaluate epidermal regeneration thickness and inflammatory cell infiltration. Masson′s Trichrome Staining: Collagen fibers were stained blue. Collagen density in the granulation tissue was quantified using Image‐Pro Plus software. Immunohistochemistry (IHC) Staining: Sections were incubated overnight at 4°C with the following primary antibodies: anti‐CD31 (Abcam, United Kingdom, 1:200 dilution), anti‐VEGFA (Affinity, United States, 1:150 dilution), and anti‐p‐SMAD2 (CST, United States, 1:100 dilution) to label vascular endothelial cells, VEGFA, and phosphorylated SMAD2 protein, respectively. The number of CD31‐positive microvessels (defined as structures containing ≥ 3 lumina) was counted to assess angiogenesis. The positive expression of VEGFA and p‐SMAD2 was observed under a microscope and semi‐quantitatively analyzed.

## 3. Statistical Analysis

Data were presented as mean ± standard deviation (SD). Statistical analysis was performed using GraphPad Prism 9.0 software. Differences among multiple groups were analyzed by one‐way analysis of variance (ANOVA) followed by Tukey′s post hoc test. A *p* value < 0.05 was considered statistically significant. All in vitro experiments were performed at least in triplicate.

## 4. Results

### 4.1. PRP Enhances Dermal Fibroblast Proliferation in a Concentration‐Dependent Manner

**Figure 1:**
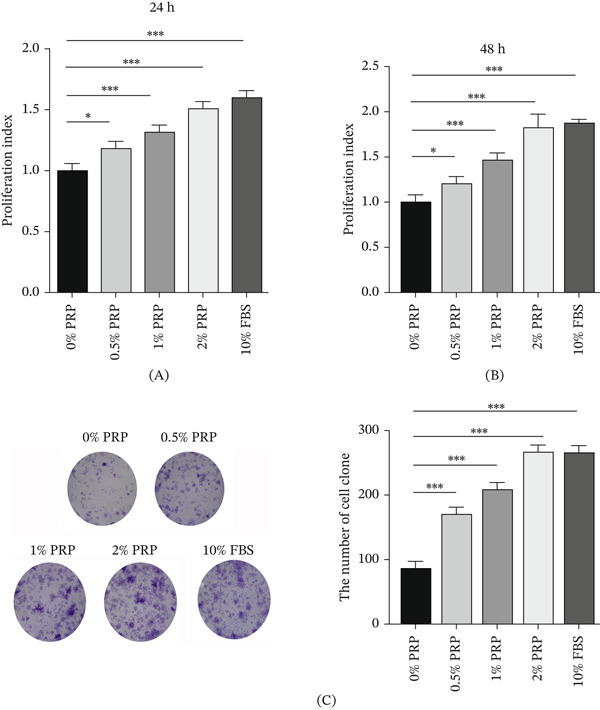
PRP enhances the proliferation of HDFs in a concentration‐dependent manner. (A and B) CCK‐8 assay showing the viability of HDFs treated with indicated concentrations of PRP (0%, 0.5%, 1%, and 2%) or 10% FBS (positive control) for 24 and 48 h. (C) Representative images (left) and quantification (right) of crystal violet‐stained HDF colonies after 14 days of culture with PRP. Colonies containing > 50 cells were counted. Data are presented as mean ± SD (*n* ≥ 3).  ^∗^ *p* < 0.05,  ^∗∗∗^ *p* < 0.001 versus control (0% PRP) group.

In order to assess the impact of PRP on the proliferative capacity of HDFs, we performed a series of in vitro experiments. The results from the CCK‐8 assay indicated that treatment with PRP at different concentrations (0.5%, 1%, and 2%) significantly promoted HDF proliferation when compared to the control group. This enhancement was observed to be both time dependent (at 24 and 48 h) and concentration dependent (Figure 1A,B). Importantly, the 2% PRP treatment group demonstrated a pro‐proliferative effect akin to that of the 10% FBS positive control, implying that PRP may have growth‐promoting capabilities comparable to traditional serum supplements.

Additionally, colony formation assays provided further evidence of the prolonged proliferative effect of PRP. Crystal violet staining revealed that PRP treatment led to a concentration‐dependent increase in both the number and size of HDF colonies. The 2% PRP group exhibited the highest efficiency in colony formation, thereby reinforcing the role of PRP in facilitating sustained self‐renewal and clonal expansion (Figure 1C).

### 4.2. PRP Stimulates DNA Synthesis in a Concentration‐Dependent Manner

**Figure 2:**
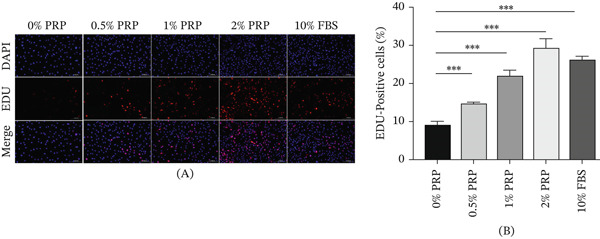
PRP stimulates DNA synthesis in HDFs. (A) Representative fluorescent images of HDFs after EdU incorporation assay following treatment with PRP or 10% FBS for 24 h. EdU‐positive nuclei (red) indicate cells in S‐phase; nuclei were counterstained with Hoechst (blue). Scale bar, 100 *μ*m. (B) Quantitative analysis of the EdU‐positive cell rate. Data are presented as mean ± SD, *n* = 3.  ^∗∗∗^ *p* < 0.001 versus control group.

To accurately evaluate the direct influence of PRP on DNA synthesis throughout cellular proliferation, an EdU incorporation assay was conducted. EdU, which serves as a thymidine analog, is integrated into newly formed DNA during the S‐phase, facilitating fluorescence‐based assessment of proliferating cells. The findings revealed that elevated concentrations of PRP were associated with an increase in the rates of EdU‐positive cells. Notably, the group treated with 2% PRP achieved a proliferation level comparable to that observed in the 10% FBS group, thereby reinforcing the pro‐proliferative effect of PRP within the cell cycle (Figure 2A,B).

These results are consistent with those obtained from the CCK‐8 assay and highlight that PRP fosters cellular proliferation by directly enhancing DNA synthesis, thereby supporting its prospective application in tissue regeneration methodologies.

### 4.3. PRP Significantly Accelerates Dermal Fibroblast Migration

**Figure 3:**
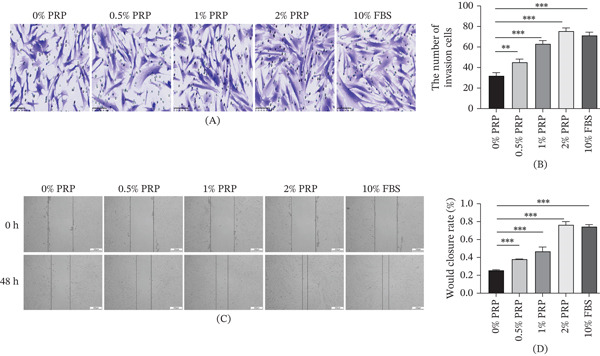
PRP promotes the migration of HDFs. (A and B) Transwell migration assay. Representative images (left) and quantification (right) of crystal violet‐stained HDFs that migrated through the membrane after 24 h of PRP treatment. (C and D) Scratch wound healing assay. Representative images (left) at 0 and 48 h postscratch and quantification (right) of the relative wound closure rate. Data are presented as mean ± SD, *n* = 3.  ^∗∗^ *p* < 0.01,  ^∗∗∗^ *p* < 0.001 versus control group.

Cell migration represents an essential biological mechanism involved in the process of wound healing, playing a pivotal role in re‐epithelialization and tissue restoration. To assess the chemotactic efficacy of PRP, we conducted Transwell migration assays. The findings revealed a significant, concentration‐dependent enhancement in the number of migrated HDFs following PRP administration, with the most pronounced effect observed in the 2% PRP group (Figure 3A,B).

To further validate these observations, we performed a scratch wound assay, which models cell migration in a two‐dimensional environment. The application of PRP notably expedited the process of wound closure in comparison to the control cohort, with the 2% PRP treatment group exhibiting the highest rate of closure (Figure 3C,D). Quantitative assessments indicated that the relative wound width in the PRP‐treated groups diminished more swiftly over time, highlighting the promigratory influence of PRP.

The consistent outcomes derived from these two complementary functional assays strongly suggest that PRP enhances both the directional and random motility of dermal fibroblasts. This stimulatory effect on cell migration further substantiates the potential of PRP as a therapeutic agent for enhancing the dynamics of wound healing.

### 4.4. PRP Promotes Collagen Synthesis

**Figure 4:**
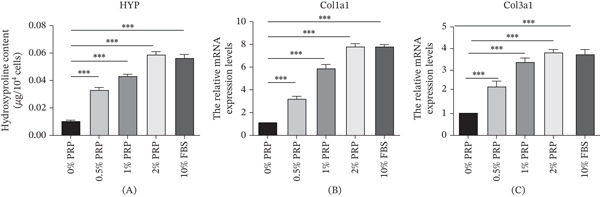
PRP promotes collagen synthesis in HDFs. (A) Quantification of hydroxyproline (HYP) content in the cell culture supernatant after PRP treatment for 48 h. (B and C) mRNA expression levels of COL1A1 and COL3A1 in HDFs treated with PRP for 24 h, as determined by qRT‐PCR. GAPDH served as the internal control. Data are presented as mean ± SD, *n* = 3.  ^∗∗∗^ *p* < 0.001 versus control group.

The remodeling of the ECM and the deposition of collagen play vital roles in the process of wound healing. In order to evaluate the influence of PRP on collagen synthesis, we initially quantified the levels of HYP, an amino acid that is a hallmark of collagen, in the supernatant of cell cultures. The data indicated that treatment with PRP resulted in a significant and concentration‐dependent elevation of HYP levels in comparison to the control group, suggesting an enhancement in overall collagen production (Figure 4A).

Furthermore, at the transcriptional level, qPCR analysis demonstrated that PRP treatment significantly and concentration dependently increased the mRNA expression of both Type I collagen (Col1a1) and Type III collagen (Col3a1) (Figure 4B,C). Taken together, these results at both protein and gene expression levels illustrate that PRP facilitates the reconstruction of the wound matrix by modulating the expression of essential ECM‐associated components.

### 4.5. PRP Activates the PI3K/AKT and TGF‐*β*/SMAD2 Signaling Pathways

In order to clarify the molecular mechanisms that contribute to the prohealing properties of platelet‐rich PRP, we conducted an investigation into the activation of pivotal signaling pathways that facilitate cellular proliferation, migration, and the synthesis of ECM components. Our primary focus was directed towards the PI3K/AKT pathway, recognized for its essential functions in enhancing cell survival and growth, alongside the TGF‐*β*/small mother against decapentaplegic homolog 2 TGF − *β*/SMAD2 pathway, which serves as a fundamental regulator of collagen synthesis and fibroblast activation.

To assess the activation of the PI3K/AKT pathway, we measured the phosphorylation levels of PI3K (p‐PI3K) and AKT (p‐AKT), as phosphorylation serves as a marker of kinase activity and subsequent signal transduction. In parallel, we evaluated the phosphorylation of SMAD2 (p‐SMAD2) and the expression levels of TGF‐*β* to monitor the activity of the TGF‐*β* signaling pathway, considering that the phosphorylation of SMAD2 is a direct consequence of TGF‐*β* receptor activation and is crucial for the transcriptional regulation of genes associated with the ECM.

**Figure 5:**
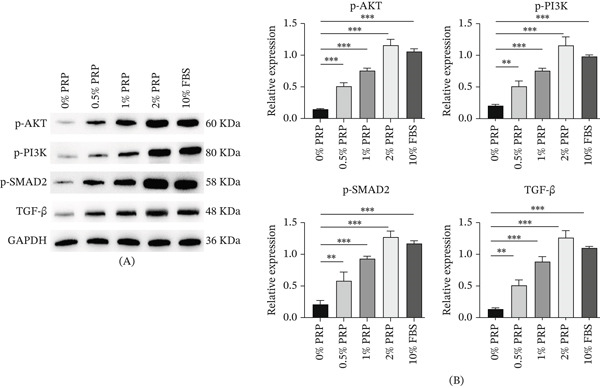
PRP activates the PI3K/AKT and TGF‐*β*/SMAD2 signaling pathways in HDFs. (A) Representative western blot images showing protein levels of p‐PI3K, p‐AKT, TGF‐*β*, p‐SMAD2, and total GAPDH (loading control) in HDFs treated with PRP for 30 min (for phospho‐proteins) or 24 h for TGF‐*β*. (B) Densitometric quantification of the protein levels normalized to GAPDH. Data are presented as mean ± SD, *n* = 3.  ^∗∗^ *p* < 0.01,  ^∗∗∗^ *p* < 0.001 versus control group.

Our western blot analyses indicated that treatment with PRP resulted in a significant concentration‐dependent increase in the protein levels of p‐PI3K, p‐AKT, p‐SMAD2, and TGF‐*β* (Figure 5A,B), thereby demonstrating the simultaneous activation of both signaling pathways. These findings imply that PRP may facilitate the healing process of diabetic wounds through the dual activation of the PI3K/AKT and TGF‐*β*/SMAD2 signaling pathways, which together promote fibroblast proliferation, migration, and collagen production.

### 4.6. PRP Accelerates Diabetic Wound Healing via the PI3K/AKT Signaling Pathway

**Figure 6:**
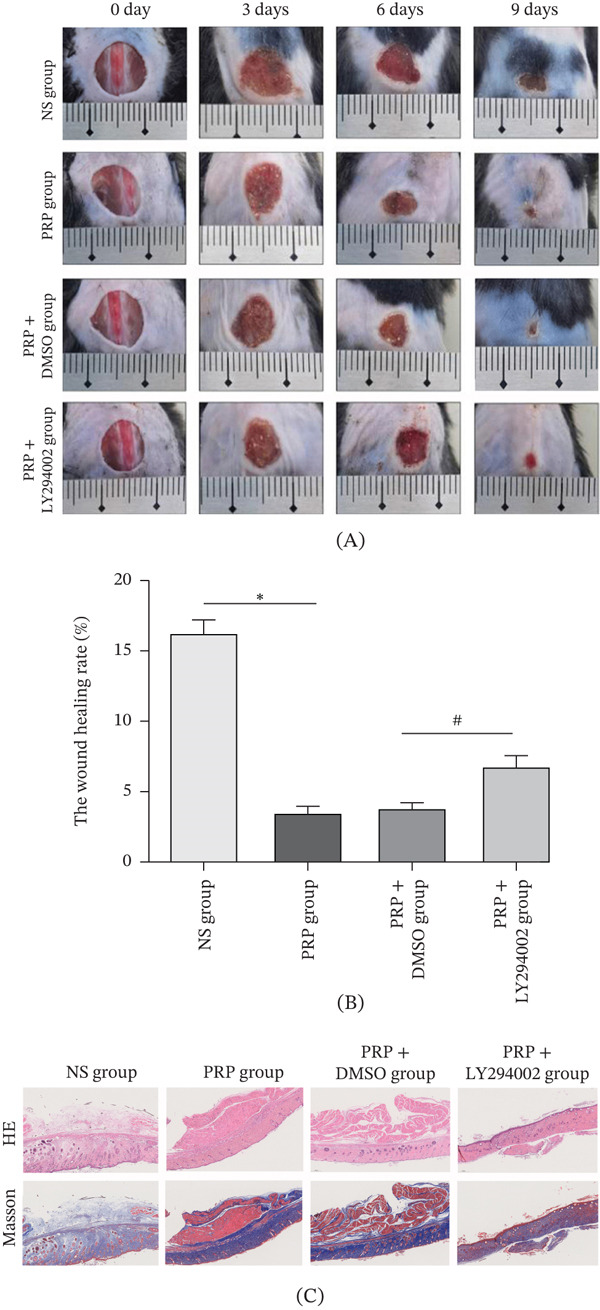
PRP accelerates diabetic wound healing and collagen deposition via the PI3K/AKT pathway in vivo. (A) Representative macroscopic images of wounds from different treatment groups (Control, PRP, PRP + DMSO, PRP + LY294002) at Days 0, 3, 6, and 9 postwounding. (B) Quantification of wound closure rate over time. (C) Representative images (left) and quantification (right) of Masson′s trichrome staining of wound tissues harvested on Day 9. Collagen fibers are stained blue. Scale bar, 50 *μ*m. Data are presented as mean ± SD, *n* = 5.  ^∗^ *p* < 0.05 versus control group, ^#^ < 0.05 versus PRP + DSMO group.

In a murine model investigating DFUs, the assessment of the wound healing dynamics indicated that the group treated with PRP exhibited a significantly accelerated healing process compared to the control group subjected to the model, as evidenced by a notably enhanced healing rate (Figure 6A,B). Conversely, the local administration of the PI3K inhibitor, LY294002, resulted in a marked attenuation of the prohealing effects attributed to PRP. In contrast, the healing outcomes observed in the PRP + DMSO (solvent control) cohort did not present a statistically significant difference when compared to the PRP‐only group (Figure 6A,B). These findings underscore the pivotal role of the PI3K/AKT signaling pathway as a fundamental mechanism through which PRP facilitates the wound healing process.

To further investigate collagen deposition and its organization, Masson′s trichrome staining was conducted on wound tissues across the different experimental groups. The PRP‐treated group demonstrated a greater abundance of collagen fibers that were not only densely packed but also well‐organized (indicated by blue staining) when compared to the model control group. Importantly, collagen deposition was significantly diminished in the PRP + LY294002 (PI3K inhibitor) group, which displayed sparse and disorganized collagen fibers (Figure 6C). This observation confirms that the inhibition of PI3K/AKT pathway activation effectively negates the procollagen synthesis effects of PRP.

Collectively, these results highlight that PRP markedly enhances collagen deposition and tissue remodeling during the healing of DFUs, and this enhancement is contingent upon the activation of the PI3K/AKT signaling pathway.

### 4.7. PRP Promotes Wound Angiogenesis via the PI3K/AKT Pathway

**Figure 7:**
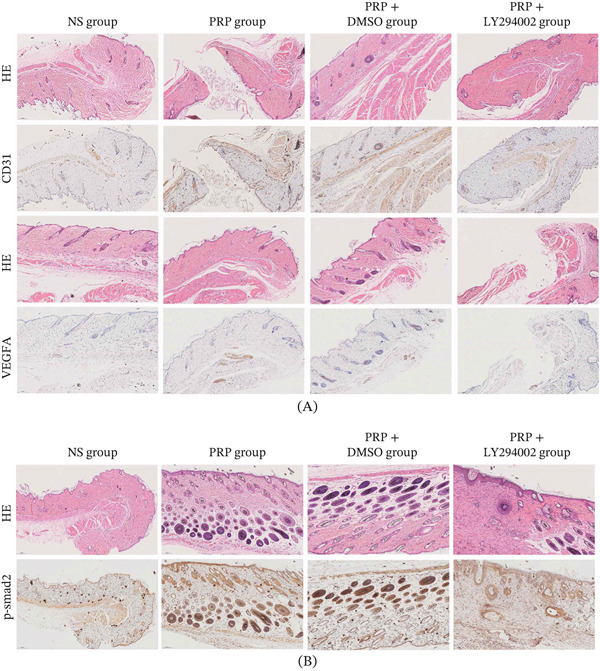
PRP promotes wound angiogenesis and cross‐regulates the TGF‐*β*/SMAD2 pathway via PI3K/AKT activation. Immunohistochemical (IHC) analysis of wound tissues on postoperative Day 9. (A) Representative IHC images of CD31‐positive microvessels and VEGF expression. (B) Representative IHC images of p‐SMAD2 expression. Scale bars, 50 *μ*m. Data are presented as mean ± SD, *n* = 5.

Angiogenesis, defined as the development of new blood vessels, plays a pivotal role in the process of wound healing, facilitating the supply of oxygen, nutrients, and immune cells to the site of injury. An immunohistochemical assessment of wound tissues conducted on postoperative Day 9 revealed a significantly elevated expression of the microvascular marker CD31 and VEGF in the granulation tissue of the group treated with PRP when compared to the model control group (Figure 7A–B). VEGF serves as a crucial regulator that promotes the proliferation and migration of endothelial cells, whereas CD31 serves as an indicator of the density of newly formed capillaries, collectively signifying an increase in angiogenic activity. Notably, in the PRP + LY294002 (a PI3K inhibitor) group, the expression levels of both CD31 and VEGF were considerably diminished (Figure 7A). This observation substantiates the hypothesis that the pro‐angiogenic effects of PRP are contingent upon the activation of the PI3K/Akt signaling pathway.

To further investigate the interplay between the PI3K/Akt and TGF‐*β*/SMAD2 pathways, we evaluated the expression of phosphorylated SMAD2 (p‐SMAD2) within the wound tissues. Immunohistochemical analysis indicated a significant increase in p‐SMAD2 expression in the PRP‐treated group, whereas its expression was markedly reduced in the PRP + inhibitor group (Figure 7B).

These collective results suggest that PRP not only stimulates the PI3K/Akt pathway to promote angiogenesis but also cross‐regulates and enhances the activation of the TGF‐*β*/SMAD2 signaling pathway. The simultaneous activation of these dual pathways likely operates synergistically to improve ECM synthesis, vascularization, and overall tissue repair, thereby providing a more thorough mechanistic understanding of the therapeutic effectiveness of PRP in the context of diabetic wound healing.

## 5. Discussion

Impaired wound healing in diabetic patients poses a considerable clinical obstacle attributable to complex cellular dysfunctions. Our research reveals that PRP significantly accelerates the healing of diabetic wounds by activating both the PI3K/AKT and TGF‐*β*/SMAD2 signaling pathways simultaneously, with the former serving as an upstream modulator of the latter. This activation of dual pathways facilitates a synergistic improvement in the functions of dermal fibroblasts, ECM remodeling, and angiogenesis.

The in vitro results furnish direct cellular evidence supporting the prohealing capabilities of PRP. The application of PRP in a concentration‐dependent manner significantly enhanced essential behaviors of HDFs that are vital for tissue regeneration, including proliferation, migration, and collagen synthesis. Notably, the effectiveness of PRP at an optimal concentration was comparable to that of 10% FBS, a commonly used growth supplement, highlighting its powerful bioactive characteristics. These findings corroborate and expand upon earlier research that documented the mitogenic and chemotactic properties of growth factors derived from PRP on various cell types involved in the wound healing process [6, 17–19]. For example, Saad et al. illustrated that PRP promotes fibroblast proliferation through the release of PDGF and TGF‐*β*, which aligns with the proliferative effects we observed [20]. However, our investigation further elucidates the concentration‐dependent nature of this response and provides a direct comparison to standard FBS supplementation.

The primary mechanistic insight of this study is the clarification of the specific signaling pathways activated by PRP and their functional hierarchy. Our findings reveal that PRP concurrently activates both the PI3K/AKT and TGF‐*β*/SMAD2 pathways in HDFs. The PI3K/AKT pathway serves as a principal regulator of cell survival, growth, and metabolism [4], whereas the TGF‐*β*/SMAD2 pathway plays a critical role in driving fibroblast activation and ECM synthesis, particularly collagen [21, 22]. This simultaneous activation is particularly significant in light of previous reports by Yang et al., which documented TGF‐*β*‐induced activation of SMAD signaling in PRP‐treated fibroblasts, yet did not explore the role of the PI3K/AKT pathway [23].

Our in vivo investigations utilizing the PI3K‐specific inhibitor LY294002 provided robust evidence underscoring the essential role of the PI3K/AKT pathway. The inhibition of this pathway not only negated the advantageous effects of PRP on wound healing, collagen accumulation, and angiogenesis but also significantly diminished the activation of the TGF‐*β*/SMAD2 pathway, as indicated by decreased levels of phosphorylated SMAD2 (p‐SMAD2) [24–26]. This pivotal finding establishes the PI3K/AKT pathway as an upstream regulator of TGF‐*β*/SMAD2 signaling within the framework of PRP action, thereby unveiling a novel mechanism of cross‐talk. While prior studies have suggested possible interactions between these pathways within cancer models, our research is the first to elucidate this hierarchical relationship in the setting of PRP‐facilitated wound healing [27]. This regulatory interaction may arise through AKT‐mediated phosphorylation of SMAD proteins, which could influence their stability, transcriptional activity, or subcellular localization [8, 9], thereby enhancing the profibrotic signaling cascade [28–30]. Consequently, our study contributes to the expanding literature that emphasizes the complex interplay between these two critical pathways in the maintenance and repair of tissue.

The simultaneous activation of the PI3K/AKT and TGF‐*β*/SMAD2 pathways by PRP establishes a synergistic network that significantly enhances the wound healing process [31]. The PI3K/AKT pathway is likely responsible for initiating cellular responses such as proliferation and migration, while simultaneously preparing and amplifying the TGF‐*β*/SMAD2‐mediated anabolic program that facilitates collagen production and tissue remodeling [32–34]. In addition, the activation of PI3K/AKT is widely recognized as a promoter of angiogenesis, primarily through the upregulation of VEGF [35–37]. Our findings, which indicate that the expression of VEGF and the formation of CD31^+^ microvessels induced by PRP are dependent on PI3K/AKT, corroborate this relationship and elucidate the augmented vascularization observed in wounds treated with PRP. Consequently, PRP functions not only as a reservoir of growth factors but also as a sophisticated biological switch that activates a complementary array of signaling cascades to address various deficiencies present in the diabetic wound environment. In summary, our investigation demonstrates that PRP accelerates the healing of diabetic wounds through a mechanism that entails the dual and interconnected activation of the PI3K/AKT and TGF‐*β*/SMAD2 signaling pathways. The PI3K/AKT pathway acts as a pivotal upstream node, essential for mediating the effects of PRP on cellular functionality, ECM synthesis, and angiogenesis, as well as for modulating the downstream activity of TGF‐*β*/SMAD2. While previous research has predominantly concentrated on isolated pathways [38], our study offers a holistic mechanistic framework that integrates these signaling axes. These results provide a solid molecular justification for the clinical application of PRP in the management of diabetic wounds and propose that approaches aimed at enhancing or replicating this dual‐pathway activation may reveal new therapeutic possibilities. Future investigations are necessary to delineate the specific receptor–ligand interactions that precede PI3K/AKT activation by PRP and to validate this mechanistic framework in human diabetic wound tissues.

## 6. Conclusions

In this study, we found that PRP facilitates diabetic wound healing by activating the PI3K/AKT pathway, which cross‐regulates TGF‐*β*/SMAD2 signaling to enhance key cellular functions for tissue regeneration. These findings provide valuable mechanistic insights into the therapeutic potential of PRP for impaired wound healing in diabetic patients.

**Nomenclature**
PRP: platelet‐rich plasma
PI3K: the phosphoinositide 3‐kinase/protein kinase B
TGF‐*β*
: transforming growth factor‐*β*
VEGF: vascular endothelial growth factor
ECM: extracellular matrix
HDFs: human dermal fibroblasts
FBS: fetal bovine serum
RT‐qPCR: reverse transcription‐quantitative PCR
IHC: immunohistochemistry
CD31: platelet endothelial cell adhesion molecule‐1
DFUs: diabetic foot ulcers

## Author Contributions

Hongyan Liu: investigation, methodology, writing – original draft, formal analysis. Wenzhen Huang: validation, methodology. Shuting Jiang: investigation, resources. Beizhan Yan: investigation, methodology, visualization. Cunquan Kong: validation, resources, project administration. Weiyan Zhu: writing – review and editing, supervision. Qi Xie: funding acquisition, supervision, writing – review and editing. Cuiyun Cui: conceptualization, funding acquisition, supervision, writing – review and editing.

## Funding

This present study was supported by grants from the National Natural Science Foundation of China (82203363) (to Qi Xie).

## Ethics Statement

This study was performed in line with the principles of the Declaration of Helsinki (1964). The use of human donor blood for the preparation of PRP was approved by the Ethics Committee of Henan Provincial People′s Hospital (Ethical approval No: 2020‐140). All donors provided written informed consent prior to blood donation. All animal experiments were approved and were conducted in accordance with the institution′s guidelines for the care and use of laboratory animals. All methods are reported in accordance with ARRIVE guidelines.

## Conflicts of Interest

The authors declare no conflicts of interest.

## Data Availability

The data that support the findings of this study are available from the corresponding author upon reasonable request.
